# Effects of Exercise on Nutritional Status in People with Cystic Fibrosis: A Systematic Review

**DOI:** 10.3390/nu14050933

**Published:** 2022-02-22

**Authors:** William B. Nicolson, Julianna Bailey, Najlaa Z. Alotaibi, Stefanie Krick, John D. Lowman

**Affiliations:** 1 Graduate Medical Education, Heersink School of Medicine, University of Alabama at Birmingham, Birmingham, AL 35294, USA; wbnicolson@uabmc.edu; 2 Division of Pulmonary, Allergy and Critical Care Medicine, University of Alabama at Birmingham, Birmingham, AL 35294, USA; juliannabailey@uabmc.edu; 3 Gregory Fleming Cystic Fibrosis Research Center, University of Alabama at Birmingham, Birmingham, AL 35294, USA; 4 Department of Physical Therapy, University of Alabama at Birmingham, Birmingham, AL 35294, USA; nzalotai@uab.edu

**Keywords:** cystic fibrosis, exercise, nutritional status, body mass index, body mass, anthropometric

## Abstract

Background: Physical exercise is an important part of regular care for people with cystic fibrosis (CF). It is unknown whether such exercise has beneficial or detrimental effects on nutritional status (body composition). Thus, the objective of this review was to evaluate the effect of exercise on measures of nutritional status in children and adults with CF. Methods: Standardized reporting guidelines for systematic reviews were followed and the protocol was prospectively registered. Multiple databases were utilized (e.g., PubMed, Scopus, and CINHAL). Two reviewers independently reviewed titles/abstracts and then the full text for selected studies. Results: In total, 924 articles were originally identified; data were extracted from 4 eligible studies. These four studies included only children; pulmonary function ranged from severe to normal, and the majority of participants were at or below their recommended weight. Exercise training did not worsen nutritional status in any study; two studies that included resistance exercise reported an increase in fat-free mass. Three of the four studies also reported increased aerobic capacity and/or muscle strength. Conclusions: Exercise training can produce positive physiologic changes in children with CF without impairing their nutritional status. In fact, resistance exercise can help improve body mass. Much less is known about how exercise may affect adults or those who are overweight.

## 1. Introduction

Cystic fibrosis (CF) is a relatively rare genetic disease affecting over 30,000 people in the United States and more than 70,000 people worldwide [1], with a prevalence varying from country to country but being as high as 1 in 900 in parts of Canada to as low as 1 in 25,000 in Finland [2]. CF is caused by a mutation in the gene responsible for the cystic fibrosis transmembrane conductance regulator (CFTR). This protein is expressed in epithelial cells and serves to directly transport chloride and indirectly affects sodium and water transport. CFTR dysfunction leads to sticky mucus, causing mucus obstruction in various organs including the lungs, pancreas, liver, and intestines. Therefore, cystic fibrosis is a multisystem disease, leading to a decreased life expectancy and significantly impaired quality of life.

CF care requires a multidisciplinary team. It not only focuses on preserving pulmonary function, but also the organ-specific and systemic manifestations of the disease as mentioned above. Malnutrition is a common problem among CF patients, and it is a consequence of multiple factors. Poor bicarbonate secretion from the pancreas, mucosal abnormalities leading to poor intestinal wall function, and poor gut transit time are all thought to contribute to decreased fat absorption [3]. Patients with more pulmonary disease manifestations have a higher concentration of circulating inflammatory markers, which has been linked to decreased fat-free mass (FFM) and bone mineral density (BMD) [4]. People with CF also have an increased resting expenditure rate at baseline [5].

Given that people with CF struggle with malnutrition, their nutritional status, assessed via anthropometric measures, most commonly body mass index (BMI, for adults) or BMI percentile (for children), is also a primary focus of CF care. BMI has been identified as an independent predictor of mortality in cystic fibrosis, with one study demonstrating a hazard ratio of 5.5 (CI 1.8–16.8) for adolescents 12 to 14 years old with a BMI of 15.8 or less [6]. BMI also has implications for morbidity in patients with CF; a cross-sectional study demonstrated decreased FEV_1_ in patients whose weight was less than 90% predicted [7]. Current CF guidelines recommend BMI goals for individuals with CF; children aged 2–20 are recommended to maintain a BMI ≥ 50th percentile, while adult women are recommended to maintain a BMI of 22–25 and adult men a BMI of 23–25 [8].

The morbidity and mortality of people with CF can also be predicted by their exercise capacity. It has been demonstrated that maximal ${\;\overset{˙}{V}}_{O_{2}}\;$ from cardiopulmonary exercise testing (CPET) can also serve as a predictor of mortality. Both Nixon et al. (1992) [9] and, more recently (2019), Hebestreit et al. [10] found a stepwise increase in survival for people with CF based on increased quantiles of percent predicted peak ${\;\overset{˙}{V}}_{O_{2}}$. Another study examined the longitudinal relationship between habitual physical activity and FEV_1_, finding that those who were more physically active had a slower decline in FEV_1_ [11].

Thus, there appears to be a potential conflict between nutritional status and exercise. Patients have an increased resting energy expenditure [5], and exercise would further increase total energy expenditure, perhaps worsening their nutritional status by causing additional weight loss. However, since patients with CF can improve their aerobic capacity through exercise, it remains unclear how exercise may affect their nutritional status/body composition. Most people think of exercise as a way to maintain or lose weight; thus, some people with CF that are underweight or at their goal weight may be reluctant to begin an exercise program. On the contrary, with the advent of highly effective modulator therapies, some patients are now concerned about gaining too much weight [12,13].

Thus, the goal of this systematic review is to help answer the question: do exercise and physical activity affect nutritional status in children and adults with cystic fibrosis? This question has clinical relevance due to the morbidity and mortality implications of malnutrition in this patient population and the perceived risk of weight loss in a population that has historically been underweight. In this new era of highly effective modulator therapies, there is now the potential risk for both normal weight obesity (increased fat mass with an otherwise normal body mass) as well as outright overweight and obese, especially as more people begin these drugs at a younger age and are on them for longer periods of time [12,13].

## 2. Materials and Methods

### 2.1. Systematic Review Design and Registration and Design

This systematic review was planned and conducted according to the Preferred Reporting Items for Systematic Reviews and Meta-Analyses guideline [14]. The protocol was registered in PROSPERO (CRD42021273303) [15].

### 2.2. Data Sources and Searches

After development of our population, intervention, comparator, outcome, and study design (PICOS) question, a medical librarian (MMB) developed a specific search strategy for multiple databases (PubMed, Scopus, Embase, CINHAL, SPORTDiscus and CENTRAL). The latest search was conducted on 20 August 2021 and all relevant records were imported into Covidence, an online software platform for conducting systematic reviews [16]. The search strategies used are in Supplementary Table S1.

### 2.3. Eligibility Criteria

Table 1 highlights the inclusion and exclusion criteria used in our review. The following nutritional status outcomes were considered: BMI, BMI percentile, BMI z-score, body mass, and fat-free mass.

### 2.4. Study Selection

Titles and abstracts of all identified articles were independently assessed, in duplicate by 3 reviewers (WBN, NZA, and JDL) using Covidence [16]. Titles and abstracts that did not provide sufficient information on the inclusion and exclusion criteria were then selected for evaluation of the full text and were included according to the eligibility criteria. Disagreements between the 2 reviewers were resolved by consultation with a third reviewer.

### 2.5. Data Extraction

Data were extracted through a standardized spreadsheet (MS Excel, Microsoft Corporation, Seattle, WA, USA) created by the authors. Extracted data included publication details, study methodology, baseline participant characteristics, intervention description, and outcomes assessed. Disagreements were also resolved by consensus. The main outcomes were anthropometrics measures (e.g., BMI and body mass). Secondary outcomes included peak ${\;\overset{˙}{V}}_{O_{2}}$ and strength.

### 2.6. Quality Assessment

The Physiotherapy Evidence Database (PEDro) scale was used to evaluate methodological quality and risk of bias of the randomized controlled trials selected in this study. The quality assessment was performed by 3 independent reviewers (NZA, SK, and JB). Any items that were unclear were rated as a “no.” Total scores were calculated based on 10 of the 11 items in the tool.

### 2.7. Data Synthesis and Analysis

Due to the heterogeneity of the outcomes collected, a meta-analysis could not be conducted. However, data extracted were quantitatively and qualitatively summarized in tabular format. BMI-for-age percentiles and weight-for-age percentiles were estimated by plotting values and extrapolating results on the National Center for Chronic Disease Prevention and Health Promotion’s growth charts [17].

## 3. Results

### 3.1. Study Search Results and Selection

**Figure 1:**
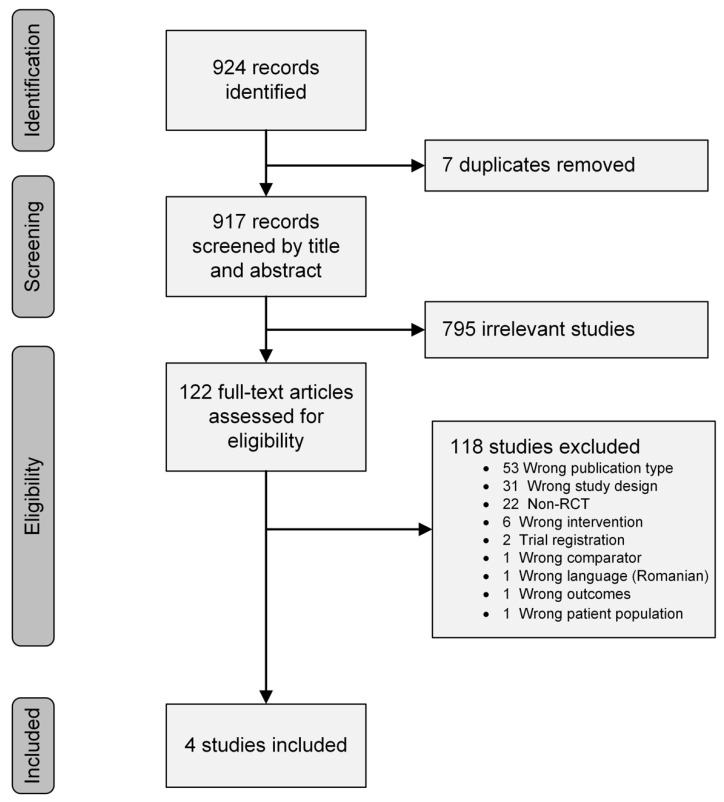
PRISMA flow diagram of the selection process and study search results.

The search strategy resulted in 924 articles, of which 122 were considered relevant for a more detailed analysis; 4 of these studies met the eligibility criteria and were included in the systematic review [18,19,20,21]. Details of the selection process, including reasons for exclusion, are illustrated in Figure 1.

### 3.2. Description of the Studies

Four studies evaluated participants’ nutritional status using BMI or other anthropometric outcomes following active exercise and compared it with a control group [18,19,20,21]. Two of these reported either BMI or BMI z-scores [18,20]; three studies reported body mass and fat-free mass [19,20,21] and one reported upper-extremity skin fold and circumference [18]. No studies were conducted in North America. All four studies included children and no study included adults. Percent predicted FEV_1_ for study participants ranged from severe to normal (Table 2), and one study reported using supplemental oxygen as needed [19]. Based on estimated mean age and body mass, 3 of the 4 studies were at or below the 25th percentile [19,20,21] and the other study was at the 50th percentile [18]; hence, almost all of the participants in the study were either normal or underweight. As recommended in a recent consensus statement [22], three used CPET results to guide the exercise prescription [19,20,21] (Table 3) and all four studies used CPET (i.e., peak ${\;\overset{˙}{V}}_{O_{2}}$) for outcome assessment (Table 4) [18,19,20,21].

Of these studies, one was performed in the hospital during an acute pulmonary exacerbation [19], two were performed in a hospital-based outpatient gym for children [20,21], and one was a home-based intervention with tele-rehab support [18] (Table 3). All four studies included aerobic exercise (AET), three at a moderate–vigorous intensity [19,20,21] and one with no specific intensity [18]. Two studies looked at AET as a separate intervention and two combined AET with resistance exercise training (RET) [20,21]. Selvadurai compared AET to RET and control [19], and Santana-Sosa’s 2014 study combined inspiratory muscle training (IMT) [23] along with AET and RET compared to a control group [21]. The primary outcome was assessed at hospital discharge (2–3 weeks) in one study [19], 8 weeks in 2 studies [20,21] and 3 months in another [18].

#### Individual Study Descriptions

The earliest RCT to investigate the effects of exercise on nutritional status (body composition) in children was Selvadurai et al. (2002) [19]. They compared AET versus RET versus a control group during hospitalization for an acute pulmonary exacerbation. All groups received “intravenous antibiotics, chest physiotherapy, and nutritional supplementation.” The respective training procedures for the exercise groups are described in Table 3. A maximal CPET using the modified Bruce protocol was used to guide the aerobic exercise prescription and as an outcome measure (peak ${\;\overset{˙}{V}}_{O_{2}}$). They also reported spirometry as an outcome. A daily 1 repetition maximum was used to guide the intensity of the resistance training group. Mean hospital length of stay for each group was ~19 days. Outcomes were assessed at discharge and 1 month post-discharge (1 month post-discharge results not shown).

Santana-Sosa et al.’s first study (2012) [20] examined a combination of AET and RET compared to a control group that only received verbal instructions regarding the benefits of exercise during an outpatient visit (Table 3). They also used results from a maximal CPET on a treadmill to guide intensity of AET (HR at the ventilatory threshold). The primary outcomes, assessed at 8 weeks, were peak ${\;\overset{˙}{V}}_{O_{2}}$ and muscle strength, but included body composition and pulmonary function as secondary outcomes.

A few years later (2014), Santana-Sosa et al. conducted a similar study [21] but included progressive IMT, using a threshold device (POWERbreathe), in addition to AET and RET (Table 3). Both of these studies also included a 4 week detraining phase (detraining results not shown).

The most recent study (2015), by Hommerding et al. [18] was a home-based intervention with tele-health support (Table 3). There was no direct supervision or reporting of adherence to the program, but, subjectively, the exercise group reported almost 4-fold more “regular physical activity”; however, only 35% of the exercise group reported exercising at least 3 days/wk, compared to 24% in the control group. Outcomes were assessed at three months. In addition to nutritional status outcomes (Table 4), they also reported spirometry and maximal CPET results (peak ${\;\overset{˙}{V}}_{O_{2}}$, exercise time, treadmill speed, and maximal HR).

### 3.3. Study Quality

All of the included studies had random allocation, baseline comparability, adequate follow up, between-group comparisons, and provided points estimates and variability (Table 5), with all scores ranging from 5 to 7. A PEDro score of 5 is considered “fair,” and scores of 6–8 are considered “good” [24]. As is typical in most exercise studies, neither the participants nor the therapists were blinded to the intervention, but two did have blinded outcomes assessors and intention-to-treat analysis [20,21].

### 3.4. Effects of Intervention

#### 3.4.1. Nutritional Status Outcomes

One study reported raw BMI scores [20] while another reported BMI z-scores [18]; BMI z-scores are measures of relative weight adjusted for child age and sex [25]. One study saw a slight decrease in BMI in both groups [20] while the other reported a slight increase [18], but neither were clinically or statistically significant, either over time or between groups (Table 4). Body mass increased overtime in the intervention and control groups in the three studies in which it was assessed [19,20,21]; in two of these studies, the increase was insignificant [20,21] but the RET group in the Selvadurai study had both statistically significant and clinically meaningful increases in body mass (7.25%), which was all due to an increase in fat-free (i.e., muscle) mass [19]. Although statistically insignificant in Santana-Sosa’s studies [20,21], fat-free mass increased in the exercise groups and decreased in the control groups.

Three of the four studies also included a follow-up period 4 weeks after the last supervised exercise session and post-training outcome assessment [19,20,21]. These results are mixed and likely the result of lack of standardization of this “detraining” period. Selvadurai reported that body mass and fat-free mass continued to rise in the aerobic training group, while body mass decreased in the resistance training group and remained stable in the control group; fat-free mass remained relatively unchanged in the resistance and control groups during the post-exercise period [19]. Santana-Sosa’s initial study reported a stable weight, BMI and fat-free mass after 4 weeks of detraining [20], but their subsequent study reported a slight, but statistically insignificant, increase in body mass (0.5 kg) [21].

#### 3.4.2. Physiologic Outcomes

Cardiorespiratory fitness, assessed as peak ${\;\overset{˙}{V}}_{O_{2}}$ from CPET, increased in the 3 studies that performed supervised AET at an appropriate intensity [19,20,21]; 2 of which reported an increase of over 20% (Table 4). It is also important to note that in spite of a potential learning effect, peak ${\;\overset{˙}{V}}_{O_{2}}$ decreased over time in the control groups of these three studies [19,20,21]. On the contrary, Hommerding did report an insignificant increase in peak ${\;\overset{˙}{V}}_{O_{2}}$ over 3 months in both the exercise and controls groups [18]. Whereas Selvadurai reported a stable or slightly higher peak ${\;\overset{˙}{V}}_{O_{2}}$ after detraining [19], both studies by Santana-Sosa reported a significant decline in peak ${\;\overset{˙}{V}}_{O_{2}}$ after cessation of regular training [20,21].

Strength was assessed in 3 studies using either an isokinetic dynomometer [19] or 5 RM on an isotonic weight machine [20,21]. Selvadurai reported an increase in lower-extremity strength of 18% in the RET but no significant change in AET or control groups [19] The studies by Santana-Sosa reported an increase in lower-extremity strength of 25 to 43% [20,21]. One study also trained and assessed inspiratory muscle performance; they reported an increase in PImax of 58% [21], which also seemed to be associated with an increase in peak ${\;\overset{˙}{V}}_{O_{2}}$ compared to their prior study (of similar design but without IMT) [20]. Surprisingly, the improvements in lower-extremity strength were preserved in all three studies that included a “detraining” phase [19,20,21]. Only Santana-Sosa’s 2014 study reported a decrease in upper-extremity strength with “detraining” [21].

## 4. Discussion

To our knowledge, this is the first review to explicitly evaluate the effect of exercise on nutritional status in individuals with CF. Other systematic reviews have focused on exercise capacity, pulmonary function, and health-related quality of life [26]. Our review yielded only four relevant RCTs [18,19,20,21], none of which included adults, and there was an overall lack of uniformity in both the interventions provided as well as the outcomes assessed.

Despite having different exercise interventions and outcomes, none of these studies reported a statistically significant decrease in FFM, BMI, body mass, or triceps skin fold thickness [18,19,20,21]. This suggests that exercise, in the short term, in spite of a population that was mostly normal to underweight, does not negatively affect body composition in CF patients. In fact, Selvadurai, whose participants were the most malnourished (mean weight for age 16%) demonstrated that RET can improve body mass, body composition and muscle strength [19]; they were also able to demonstrate that AET led to larger increases in aerobic capacity and a slight, but statistically insignificant, increase in body mass compared to the control group; they ultimately suggested that a combined training program may be of most benefit to patients with CF. In their initial study (2012) [20], Santana Sosa did not notice any significant difference in BMI or FFM with a combination of AET and RET, but in their later study (2014) [21], they did find a significant increase in FFM in the exercise training group.

The mixed results of these studies may be due to multiple factors. They all used different exercise methods in their studies. In addition, they have a limited sample size in their study populations, with the largest study having 66 people [19]. Moreover, they only examined the results of their intervention over short periods of time, the longest being three months [18]. No RCTs were identified that examined changes in nutritional status related to exercise over long periods of time in a CF patient population. Since their exercise regimens were tightly controlled by either hospital admission or frequent phone calls, the lack of detrimental effects from exercise in these studies is likely a reliable outcome, despite variations in benefit. This lends credibility into studying the effects of exercise on body composition in CF patients over the long term. However, given the positive benefits of exercise on other parameters, assigning participants to a prolonged control group could be considered unethical.

Our results in children are similar to those found by Elice et al. in a matched cohort study of adults; they found that only 24% of those that exercised regularly had an altered BMI compared to 41% in those that did not exercise regularly [27]. More recently (2021), Van Biervliet reported on a prospective pre–post intervention study design for patients with CF (6 to 40 years old) to improve nutritional status and body composition; patients participated in a short-term (3 weeks), inpatient, physical exercise and nutritional intervention program [28]. Weight, BMI, and fat-free mass were improved in both children and adults; in addition, the number of adults classified as “malnourished” decreased from 41% to 24%, but was unchanged (24%) in children. To our knowledge, the largest exercise-related CF study was recently completed and published [29]. The ACTIVATE-CF study randomized 117 children (≥12 years old) and adults to a 12 month partially supervised vigorous physical activity intervention [29]; although data on body composition were collected as a secondary outcome [30], these data are not yet reported.

Thus, based on the data we found in these four RCTs of exercise interventions in children with CF, as well as other non-RCT studies, there is no evidence, even in normal to underweight patients, that either AET or RET will worsen an individual’s nutritional status; in fact, RET could help maintain or increase body mass and potentially lean body mass. Clinicians should counsel patients that are concerned about the speculative effects of exercise on their nutritional status and body composition that exercise is not detrimental and may even improve their nutritional status. The CF care team should continue to rely on the CF care team’s registered dietitian to provide appropriate individualized nutrition care plans that compliment exercise regimens to help patients meet their personal goals related to weight and body composition (e.g., the team reported by Van Biervliet included a physician, dietician, psychologist, social worker and physical therapist [28]). In addition, both AET and RET have additional benefits for patients with CF (increased aerobic capacity and strength), benefits which are associated with a positive prognosis.

Hommerding demonstrated an increase in physical activity level in patients that had frequent follow-up for their exercise regimen [18]. For those working in multidisciplinary settings, referral to a physical therapist or an exercise specialist with experience with CF that can guide exercise regimens over time and as their health waxes and wanes would be of more benefit. There are standard guidelines on exercise testing [22], exercise prescription [31], and physical activity assessment [32] for clinicians working with individuals with CF.

We were surprised at the lack of high-quality RCTs that included nutritional status as an outcome of exercise interventions. Future research should not only include longer-term outcomes of exercise training on body composition in patients with CF, but should include adults as well as differentially look at the impact of exercise on patients that are underweight, normal weight and overweight, and should include alternatives to BMI and body mass in assessing nutritional status (e.g., dual-energy X-ray absorptiometry, skinfold thickness, bioelectrical impedance, and peripheral quantitative computed tomography) [12,33].

## Figures and Tables

**Table 1:** Summary of inclusion and exclusion criteria based on population/patient, intervention, comparator, outcome, and study design (PICOS).

PICOS Parameter	Inclusion Criteria	Exclusion Criteria
Population	Children and adults with cystic fibrosis (underweight, normal weight, or overweight)	Infants, toddlers and preschoolers (<5 years old)
Intervention	Exercise or physical activity	Passive exercise (e.g., stretching, range of motion)
Comparison	Non-exposed control group	
Outcome	Body mass index, body mass, body composition (e.g., fat-free mass)	
Study design	Randomized controlled trials	Language other than English, German, Spanish, or French

**Table 2:** Study site and baseline population demographics (sex, age, pulmonary function and nutritional status) for all participants.

Ref.	First Author	Year	State/Country	Number of Participants (Female)	Age	FEV_1_%	Body Mass (kg)	Weight for Age% ^†^	BMI (kg/m^2^)	BMI for Age% ^§^
[19]	Selvadurai, H.C.	2002	New South Wales, Australia	66 (38)	13 (2)	57 (17)	38.0 (7.8)	16th	NA	NA
[20]	Santana-Sosa, E.	2012	Madrid, Spain	22 (9)	10.5 (2)	83 (11) *	37.0 (3.0)	65th	17.8	61st
[21]	Santana-Sosa, E.	2014	Madrid, Spain	20 (8)	10.5 (1)	73 (9) *	34.0 (3.8)	47th	16.1	34th
[18]	Hommerding, P.X.	2015	Rio Grande do Sul, Brazil	34 (14)	13 (3)	98 (20)	45.6 (15.3)	50th	NA	NA

Values reported as the mean (standard deviation) of combined intervention and control groups (NA: not available based on data provided). FEV_1_%: percent predicted of forced expiratory volume in 1 s; BMI: body mass index. * Estimated values based on raw FEV (L/s) and other data reported in manuscript. ^†^ Estimated weight-for-age percentile based on reported sex proportion and mean age and body mass for all participants. ^§^ Estimated based on sex, age, and reported BMI using standardized growth charts.

**Table 3:** Characteristics of intervention setting and groups.

Ref.	Setting (Duration)	Exercise Group(s)		Control Group
[19]	Acute/ Inpatient (~2–3 wks)	**Aerobic exercise training**	**Resistance exercise training**	No exercise
		*Mode*: treadmill or stationary cycling	*Mode:* Isotonic weight machines	
		*Intensity*: 70% of HR_peak_	*Intensity*: 70% 1 RM	
		*Duration*: 30 min	*Duration*: 5 sets of 10 repetitions	
		*Frequency*: 5 d/wk	*Frequency*: 5 d/wk	
		*Other*: Supplemental oxygen was titrated to keep SpO_2_ > 90% (if needed). Training was stopped if dyspnea ≥ 7 on Borg CR10 scale. Each session was individually supervised	*Other*: Upper- and lower-extremity exercises (specific exercises and number not defined). Each session was individually supervised	
[20]	Hospital-based, outpatient gym (8 weeks)	**Aerobic exercise training**		Chest physiotherapy twice daily and provided verbal instruction on the benefits of physical activity
		*Mode*: Cycle ergometer		
		*Intensity*: HR at ventilatory threshold (determined during exercise test)		
		*Duration*: 20–40 min		
		*Frequency*: 3 d/wk		
		*Other*: HR monitor was worn during aerobic exercise. 10 min warmup on cycle. Each session was individually supervised		
		**Resistance exercise training**		
		*Mode*: Isotonic weight machines (bench press, shoulder press, leg extension, leg press, leg curl, abdominal crunch, low back extension, arm curl, elbow extension, seated row, and lateral pulldown)		
		*Intensity*: Progressive, from 40 to 60% of 5 RM		
		*Duration*: 3 circuits of 1 set of 12–15 repetitions of each exercise		
		Frequency: 3 d/wk (following aerobic exercise session)		
[21]	**Hospital-based, outpatient gym** **(8 weeks)**	**Aerobic exercise**		Chest physiotherapy twice daily, IMT at 10% of PI_max_, and provided instruction on the benefits of physical activity
		*Mode*: Cycle ergometer and “active playing” (i.e., running and soccer)		
		*Intensity*: HR at ventilatory threshold (determined during exercise test)		
		*Duration*: 20–40 min		
		*Frequency*: 3 d/wk		
		*Other*: HR monitor was worn during aerobic exercise. 10 min warmup on cycle. Each session was individually supervised		
		**Resistance exercise**		
		*Mode*: isotonic weight machine (leg press, pull down, leg extension, bench press, leg curl, seated row and abdominal crunch)		
		*Intensity*: Progressive, beginning at 50% of 5 RM		
		*Duration*: 3 circuits of 1 set of 12–15 repetitions of each exercise		
		*Frequency*: 3 d/wk (following aerobic exercise)		
		**Inspiratory muscle training**		
		*Mode*: PowerBreathe threshold loading device		
		*Intensity*: 40–50% of PI_max_.		
		*Duration*: 30 breaths		
		*Frequency*: twice daily, 6–7 d/wk		
		*Other*: One IMT session was performed during the 3 d/wk supervised sessions; the remaining IMT sessions were performed independently at home		
[18]	Home-based with tele-health follow-up every 2 weeks (3 months)	**Aerobic exercise**		Verbal instructions regarding aerobic exercise which are part of routine outpatient care
		*Mode*: Self-selected per recommendations (e.g., walking, jogging, swimming, dancing skipping rope)		
		*Intensity*: No recommendations given		
		*Duration*: ≥20 min		
		*Frequency*: at least 2 d/wk		
		*Other*: Written manual of aerobic and stretching exercises provided		

Abbreviations: HR = heart rate; HRpeak = peak HR; SpO_2_ = pulse oximetry saturation; Borg CR10 = Borg category ratio 10 scale; 1 RM = 1 repetition maximum; 5 RM = 5 repetition maximum; PImax = maximal inspiratory pressure; IMT = inspiratory muscle training.

**Table 4:** Nutritional status and physiologic outcomes of randomized controlled trials of exercise in people with CF.

Ref.	Nutritional Status Outcomes		Physiologic Outcomes	Conclusions
	BMI	Other		
[19]	Not reported/unable to calculate based on data reported	∆ body mass (kg):	∆ VO_2 peak_ (mL/kg/min):	AET improved body composition (2%) and peak VO_2_ (22%) RET improved body composition (7%) and was the only group to improve LE strength (18%) The CTL group had an improvement in body mass (2.7%), but an insignificant loss of strength and aerobic capacity
		AET ↑ 0.80 (0.64) *	AET ↑ 7.3 (6.3) *	
		RET ↑ 2.76 (0.70) *	RET ↑ 0.7 (5.9)	
		CTL ↑ 1.03 (0.58) *	CTL ↓ 1.2 (6.2)	
		∆ fat-free mass (kg):	∆ strength (Nm):	
		AET ↑ 0.61 (0.37) *	AET ↑ 1.8 (6.2)	
		RET ↑ 2.40 (0.46) *	RET ↑ 18.3 (7.0) *	
		CTL ↑ 0.60 (0.32) *	CTL ↓ 6.3 (6.1)	
[20]	∆ BMI (kg/m^2^): ET ↓ 0.1 CTL ↓ 0.1	∆ body mass	∆ VO_2 peak_	No significant changes in body composition variables. Peak VO_2_ improved ~10% and strength ~25% in the ET group compared to a ~6% decrease in peak VO_2_ and −2 to +5% change in strength of the CTL group
		(kg):	(mL/kg/min):	
		ET ↑ 0.6	ET ↑ 3.9 (2–6) *	
		CTL ↑ 1.1	CTL ↓ 2.2 (−5–0)	
		∆ fat-free mass (%):	∆ strength (kg):	
		ET ↑ 1.3	ET ↑ 10.5 (7–14) *	
		CTL ↓ 0.2	CTL not reported	
[21]	Not reported/unable to calculate based on data reported	∆ body mass	∆ VO_2 peak_ (mL/kg/min):	No significant changes in body mass, but fat-free mass increased in the ET group. Peak VO_2_, LE strength, and inspiratory muscle strength increased 22%, 43%, and 58%, respectively, in the ET group. There were no significant changes in the CTL group
			ET ↑ 6.9 *	
		(kg):	CTL ↓ 0.6	
		ET ↑ 1.4	∆ strength (kg):	
		CTL ↑ 0.9	ET ↑ 27 *	
		∆ fat-free mass (%):	CTL ↓ 1.3	
		ET ↑ 1.0	∆ PI_max_ (mm Hg):	
		CTL ↓ 0.1	ET ↑ 39 *	
			CTL ↑2.3	
[18]	∆ BMI z-score: ET ↑ 0.2 (0.5) CTL ↑ 0.1 (0.2)	∆ Triceps skin fold	∆ VO_2 peak_	In spite of self-reported increase in regular physical activity, there were no significant changes in any outcome measures in either group
		ET ↑ 0.3 (1.3)		
		CTL ↓ 0.1 (1.0)	(mL/kg/min):	
		∆ Arm muscle circ. (cm)	ET ↑ 1.1 (4.6)	
		ET ↑ 0.1 (0.4)	CTL ↑ 2.3 (11.9)	
		CTL ↓ 0.1 (0.2)		

Abbreviations: AET = aerobic exercise training group; RET = resistance exercise training group; ET = exercise training group; CTL = control group; ∆ = change; ↑ = increase; ↓ = decrease; circ. = circumference. Data are presented as either the mean (SD) or the mean (95% CI); * indicates a significant change (*p* < 0.05).

**Table 5:** Methodologic quality and statistical reporting assessment, using the PEDro scale, of randomized controlled trials evaluating the effect of exercise on anthropometric outcomes in people with CF.

PEDro Criteria	Selvadurai 2002 [19]	Santana-Sosa 2012 [20]	Santana-Sosa 2014 [21]	Hommerding 2015 [18]
1—Eligibility criteria	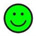	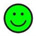	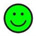	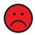
2—Random allocation	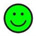	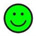	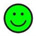	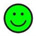
3—Concealed allocation	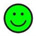	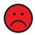	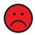	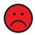
4—Baseline comparability	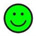	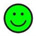	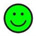	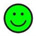
5—Blind subjects	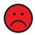	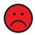	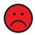	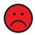
6—Blind therapists	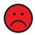	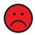	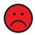	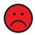
7—Blind assessors	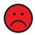	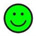	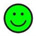	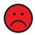
8—Adequate follow-up	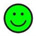	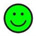	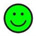	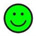
9—Intention-to-treat analysis	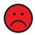	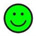	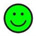	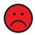
10—Between-group comparisons	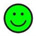	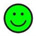	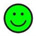	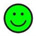
11—Points estimates and variability	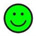	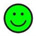	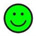	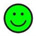
Total score	6	7	7	5

Note: Scores range from 0 to 10. Eligibility criteria (item 1) do not contribute to the total score. 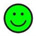 indicates criteria was fulfilled; 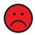 indicates criteria was not met.

## Supplementary Materials

The following supporting information can be downloaded at: https://www.mdpi.com/article/10.3390/nu14050933/s1, Table S1. Database search strategies.
