# Supplementary material for: Effects of Exercise on Nutritional Status in People with Cystic Fibrosis: A Systematic Review
Source: Nutrients. 2022 Feb 22;14(5):933. doi: 10.3390/nu14050933 (PMC8912872; doi:10.3390/nu14050933)
Supplement: Supplementary file 1 [file nutrients-14-00933-s001.zip › nutrients-1566141-supplementary-done-done.pdf]

nutrients MDPI

Supplementary Table S1. Database search strategies

Database	Search Terms
PubMed	(("Cystic Fibrosis"[Mesh] OR "Cystic Fibrosis" [tiab] OR Mucoviscidosis [tiab] OR Fibrocystic-Disease* [tiab]) AND ("Exercise"[Mesh] OR Exercise* [tiab] OR "Exercise Therapy"[Mesh] OR "Physical Exertion"[Mesh] OR Physical-Exertion* [tiab] OR physical-effort* [tiab] OR physical-activit*[Title/Abstract] OR Gymnastic*[Title/Abstract] OR Calisthenic*[Title/Abstract] OR stretch*[Title/Abstract] OR train*[Title/Abstract] OR run [Title/Abstract] OR runs [tiab] OR run-ning [tiab] OR jog [Title/Abstract] OR jogs [tiab]OR jogging [tiab] OR swim*[Title/Abstract] OR walk*[Title/Abstract] OR climb*[Title/Abstract] OR weight-lift*[Title/Abstract] OR Ambulat*[Ti-tle/Abstract] OR Physical-Exertion[Title/Abstract] OR physical-effort[Title/Abstract] OR Fit-ness[Title/Abstract] OR pilate*[Title/Abstract] OR qigong[Title/Abstract] OR qi- gong[Title/Ab-stract] OR ch'i-kung[Title/Abstract] OR danc*[Title/Abstract] OR tai- ji[Title/Abstract] OR tai-chi[Title/Abstract] OR tai-ji-quan[Title/Abstract] OR taiji[Title/Abstract] OR taijiquan[Title/Ab-stract] OR t'ai-chi[Title/Abstract] OR tai-chi- chuan[Title/Abstract] OR yoga[Title/Abstract] OR Sport*[Title/Abstract] OR athletic*[Title/Abstract] OR baseball*[Title/Abstract] OR softball*[Ti-tle/Abstract] OR basketball*[Title/Abstract] OR netball*[Title/Abstract] OR bicycling[Title/Ab-stract] OR cycling[Title/Abstract] OR boxing*[Title/Abstract] OR cricket[Title/Abstract] OR foot-ball*[Title/Abstract] OR rugb*[Title/Abstract] OR golf*[Title/Abstract] OR hockey*[Title/Abstract] OR wrestl*[Title/Abstract] OR martial-art*[Title/Abstract] OR hap-di-do[Title/Abstract] OR judo[Title/Abstract] OR karate[Title/Abstract] OR jujitsu[Title/Abstract] OR tae-kwon-do[Title/Ab-stract] OR aikido[Title/Abstract] OR wushu[Title/Abstract] OR kung-fu[Title/Abstract] OR gong-fu[Title/Abstract] OR gongfu[Title/Abstract] OR mountaineer*[Title/Abstract] OR tennis[Title/Ab-stract] OR Racquetball[Title/Abstract] OR Racketball[Title/Abstract] OR Racket-Ball[Title/Ab-stract] OR Badminton[Title/Abstract] OR Lacrosse[Title/Abstract] OR skating*[Title/Abstract] OR skateboard*[Title/Abstract] OR snowmobiling[Title/Abstract] OR sledding[Title/Abstract] OR ski-ing[Title/Abstract] OR snowboard*[Title/Abstract] OR soccer*[Title/Abstract] OR track*[Title/Ab-stract] OR volleyball*[Title/Abstract] OR surfing[Title/Abstract] OR rowing[Title/Abstract] OR polo[Title/Abstract] OR kayaking[Title/Abstract] OR canoeing[Title/Abstract] OR boating[Ti-tle/Abstract] OR surfboarding[Title/Abstract] OR recreation*[Title/Abstract] OR garden*[Title/Ab-stract] ORballet[Title/Abstract] OR hip-hop[Title/Abstract] OR jazz[Title/Abstract] OR tap[Ti-tle/Abstract] OR salsa[Title/Abstract] OR "Sports"[Mesh] OR "Recreation"[Mesh]) AND ("Physi-cal Appearance, Body"[Mesh] OR physical-appearance [tiab] OR outward- appearance [tiab] OR "Body Composition"[Mesh] OR "Body Composition" [tiab] OR ("Body Weights and Measures"[Mesh] NOT "Organ Size"[Mesh]) OR body-measure* [tiab] OR Body-weight [tiab] OR body-mass-index [tiab] OR waist-circumference [tiab] OR weight-loss [tiab] OR weight-gain [tiab])) NOT ("animals"[MeSH Terms] NOT "humans"[MeSH Terms])<br>refs 253

Scopus	TITLE-ABS (exercise* OR "Physical Exertion*" OR "physical effort*" OR "physical activit*" OR gymnastic* OR calisthenic* OR stretch* OR train* OR run OR runs OR running OR jog OR jogs OR jogging OR swim* OR walk* OR climb* OR "weight lift*" OR ambulat* OR fitness OR pilate* OR qigong OR qi-gong OR ch'i-kung OR danc* OR tai-ji OR tai-chi OR tai-ji-quan OR taiji OR taijiquan OR "t'ai-chi" OR "tai-chi-chuan" OR yoga OR sport* OR athletic* OR baseball* OR softball* OR basketball* OR netball* OR bicycling OR cycling OR boxing* OR cricket OR football* OR rugb* OR golf* OR hockey* OR wrestl* OR "martial-art*"OR "hap-di-do" OR judo OR karate OR jujitsu OR "tae-kwon-do" OR aikido OR wushu OR "kung-fu" OR "gong-fu" OR gongfu OR mountaineer* OR tennis OR racquet-ball OR racketball OR {Racket Ball} OR badminton OR lacrosse OR skating* OR skateboard* OR snowmobiling OR sledding OR skiing OR snowboard* OR soccer* OR track OR volleyball OR surf-ing OR rowing OR polo OR kayaking OR canoeing OR boating OR surfboarding OR recreation* OR garden* OR ballet OR hip-hop OR jazz OR tap OR salsa) AND TITLE-ABS ({Cystic Fibrosis} OR mucoviscidosis OR "Fibrocystic Disease*") AND TITLE-ABS ({physical appearance} OR {physical appearances} OR {outward appearance} OR {outward appearances} OR {Body Composition} OR {body measure} OR {body measures} OR {Body weight} OR {body mass index} OR {waist circumference} OR {weight loss} OR {weight gain})<br><br>refs 188
Embase	(('cystic fibrosis'/exp OR ((cystic NEXT/2 fibrosis):ti,ab) OR mucoviscidosis:ti,ab OR 'fibrocystic dis-ease*':ti,ab OR 'cystic disease':ti,ab OR 'pancreas fibrosis':ti,ab OR 'pancreatic fibrosis':ti,ab) AND ('body size'/exp OR 'body mass'/exp OR 'body weight'/exp OR 'body composition'/exp OR 'physical appear-ance'/exp OR 'physical appearance':ti,ab OR 'outward appearance':ti,ab OR 'body composition':ti,ab OR 'body measure*':ti,ab OR 'body weight':ti,ab OR 'body mass index':ti,ab OR 'waist circumference':ti,ab OR 'weight loss':ti,ab OR 'weight gain':ti,ab) AND (exercise*:ti,ab OR 'physical activit*':ti,ab OR gymnas-tic*:ti,ab OR calisthenic*:ti,ab OR run*:ti,ab OR jog*:ti,ab OR swim*:ti,ab OR walk*:ti,ab OR climb*:ti,ab OR 'weight lift*':ti,ab OR pilate*:ti,ab OR qigong:ti,ab OR 'qi gong':ti,ab OR danc*:ti,ab OR 'tai ji':ti,ab OR 'tai chi':ti,ab OR 'tai ji quan':ti,ab OR taiji:ti,ab OR taijiquan:ti,ab OR 'tai chi chuan':ti,ab OR yoga:ti,ab OR sport*:ti,ab OR athletic*:ti,ab OR baseball:ti,ab OR softball:ti,ab OR basketball:ti,ab OR netball:ti,ab OR bicycling:ti,ab OR cycling:ti,ab OR boxing:ti,ab OR cricket:ti,ab OR football:ti,ab OR rugb*:ti,ab OR golf*:ti,ab OR hockey*:ti,ab OR wrestl*:ti,ab OR 'martial art*':ti,ab OR 'hap di do':ti,ab OR judo:ti,ab OR karate:ti,ab OR jujitsu:ti,ab OR 'tae kwon do':ti,ab OR aikido:ti,ab OR wushu:ti,ab OR 'kung fu':ti,ab OR 'gong fu':ti,ab OR gongfu:ti,ab OR mountaineer*:ti,ab OR tennis:ti,ab OR racquetball:ti,ab OR racket-ball:ti,ab OR 'racket ball':ti,ab OR badminton:ti,ab OR lacrosse:ti,ab OR skating*:ti,ab OR skate-board*:ti,ab OR snowmobiling:ti,ab OR sledding:ti,ab OR skiing:ti,ab OR snowboard*:ti,ab ORsoccer:ti,ab OR track:ti,ab OR volleyball:ti,ab OR surfing:ti,ab OR rowing:ti,ab OR polo:ti,ab OR kayaking:ti,ab OR ca-noeing:ti,ab OR boating:ti,ab OR surfboarding:ti,ab OR recreation*:ti,ab OR ballet:ti,ab OR 'hip hop':ti,ab OR jazz:ti,ab OR tap:ti,ab OR salsa:ti,ab OR fitness:ti,ab OR 'exercise'/exp OR 'sport'/exp OR 'recrea-tion'/exp OR 'fitness'/exp OR 'physical activity'/exp) NOT ('animals'/exp NOT 'humans'/exp)) AND ('arti-cle'/it OR 'conference abstract'/it OR 'conference paper'/it OR 'review'/it)<br><br>refs 680

CINAHL	((MH "Exercise+") OR (MH "Sports+") OR (MH "Recreation+") OR (MH "Physical Fitness+") OR (MH "Physical Activity") OR TI (Exercise* OR physical-activit* OR Gymnastic* OR Calisthenic* OR run* OR jog* OR swim* OR walk* OR climb* OR weight- lift* OR pilate* OR qigong OR qi-gong OR ch'i-kung OR danc* OR tai-ji OR tai-chi OR tai-ji-quan OR taiji OR taijiquan OR t'ai-chi OR tai-chi-chuan OR yoga OR Sport* OR athletic* OR baseball* OR softball* OR basketball* OR netball* OR bicycling OR cycling OR boxing* OR cricket OR football* OR rugb* OR golf* OR hockey* OR wrestl* OR martial-art* OR hap-di-do OR judo OR karate OR jujitsu OR tae-kwon-do OR aikido OR wushu OR kung-fuOR gong-fu OR gongfu OR mountaineer* OR tennis OR Racquetball OR Racketball OR Racket-Ball OR Badminton OR Lacrosse OR skating* OR skateboard* OR snowmobiling OR sledding OR skiing OR snowboard* OR soccer OR track OR volleyball OR surfing OR rowing OR polo OR kayaking OR canoeing OR boating OR surfboarding OR recreation* OR ballet OR hip-hop OR jazz OR tap OR salsa OR fitness) OR AB (Exercise* OR physical-activit* OR Gymnastic* OR Calisthenic* OR run* OR jog* OR swim* OR walk* ORclimb* OR weight-lift* OR pilate* OR qigong OR qi-gong OR ch'i-kung OR danc* OR tai-ji OR tai-chi OR tai-ji-quan OR taiji OR taijiquan OR t'ai-chi OR tai-chi-chuan OR yoga OR Sport* OR athletic* OR baseball* OR softball* OR basketball* OR netball* OR bicycling ORcycling OR boxing* OR cricket OR football* OR rugb* OR golf* OR hockey* OR wrestl* ORmartial-art* OR hap-di-do OR judo OR karate OR jujitsu OR tae-kwon-do OR aikido OR wushu OR kung-fu OR gong-fu OR gongfu OR mountaineer* OR tennis OR Racquetball OR Racketball OR Racket-Ball OR Badminton OR Lacrosse OR skating* OR skateboard* OR snowmobiling OR sledding OR skiing OR snowboard* OR soccer OR track OR volleyball OR surfing OR rowing OR polo OR kayaking OR canoeing OR boating OR surfboarding OR recreation* OR ballet OR hip-hop OR jazz OR tap OR salsa OR fitness)) AND ((MH "Cystic Fibrosis") OR TI ("Cystic Fibrosis" OR Mucoviscidosis OR fibrocystic- disease*) OR AB (Mucoviscidosis OR fibrocystic-disease* OR "Cystic Fibrosis")) AND ((MH "Body Constitution+") OR (MH "Body Composition+") OR (MH "Personal Appearance") OR TI (physical-appearance OR outward-appearance OR "Body Composition" OR body-measure* OR Body-weight OR body-mass-index OR waist-circumference OR weight-loss OR weight-gain) OR AB (physical-appearance OR outward-appearance OR "Body Composition" OR body-measure* OR Body-weight OR body-mass-index OR waist-circumference OR weight-loss OR weight-gain)) refs 78
SPORTDiscus	(DE "CYSTIC fibrosis" OR TI ("CYSTIC fibrosis" OR mucoviscidosis OR Fibrocystic- Disease*) OR AB ("CYSTIC fibrosis" OR mucoviscidosis OR Fibrocystic-Disease*)) AND(DE "BODY weight" OR DE "LEANNESS" OR DE "OBESITY" OR DE "BODY mass index" OR DE "BODY composition" OR DE "ALCOHOL in the body" OR DE "AMINES in the body" OR DE "AMINO acids in the body" OR DE "ARSENIC in the body" OR DE "BONE densitometry" OR DE "BONE density" OR DE "CALCIUM in the body" OR DE "CARBOHYDRATES in the body" OR DE "CARBON dioxide in the body" OR DE "CARBON in the body" OR DE "CHLORIDES in the body" OR DE "CHOLESTEROL in the body" OR DE "CHROMIUM in the body" OR DE "CITRIC acid in the body" OR DE "COBALT in the body" OR DE "COPPER in the

	body" OR DE "HUMAN body composition" OR DE "IODINE in the body" OR DE "IRON in the body" OR DE "LACTOSE in the body" OR DE "LEAD in the body" OR DE "LIPIDS in the body" OR DE "MAGNESIUM in the body" OR DE "MERCURY in the body" OR DE "METALS in the body" OR DE "MINERALS in the body" OR DE "NITROGEN in the body" OR DE "OXYGEN in the body" OR DE "PHENOLS in the body" OR DE "PHOSPHORUS in the body" OR DE "POTASSIUM in the body" OR DE "PROTEINS in the body" OR DE "SALT in the body" OR DE "SILICON in thebody" OR DE "SODIUM in the body" OR DE "SUGAR in the body" OR DE "SULFUR in the body" OR DE "TYROSINE in the body" OR DE "VITAMIN A in the body" OR DE "VITAMIN D in the body" OR DE "ZINC in the body" OR DE "WEIGHT gain" OR DE "WEIGHT loss" OR DE "BODY image" OR DE "BODY image in girls" OR DE "BODY image in women" OR DE "PHYSICAL characteristics (Human body)" OR TI (physical-appearance OR outward- appearance OR "Body Composition" OR body-measure* OR Body-weight OR body-mass- index OR waist-circumference OR weight-loss OR weight-gain) OR AB (physical- appearance OR outward-appearance OR "Body Composition" OR body-measure* OR Body-weight OR body-mass-index OR waist-circumference OR weight-loss OR weight-gain)) AND (DE "TAI chi" OR DE "BOXING" OR DE "RECREATION" OR DE "FAMILY recreation" OR DE "INDUSTRIAL recreation" OR DE "OUTDOOR recreation" OR DE "PLAY" OR DE "RECREATION for older people" OR DE "RECREATION programs" OR DE "RECREATIONAL sports" OR DE "RECREATIONAL therapy" OR DE "SCHOOL exercises & recreations" OR DE "SPORTS" OR DE "STUDENT recreation" OR DE "WILDLIFE-related recreation" OR DE "YOUTH recreation" OR DE "MARTIAL arts" OR DE"JUDO" OR DE "KARATE" OR DE "JIU-jitsu" OR DE "TAE kwon do" OR DE "AIKIDO" OR DE "CHINESE martial arts" OR DE "TENNIS" OR DE "RACQUETBALL" OR DE "BADMINTON (Game)" OR DE "LACROSSE" OR DE "SKATING" OR DE "BARREL jumping" OR DE "FIGURE skating" OR DE "FREE skating" OR DE "ICE dancing" OR DE "ICE sledding" OR DE "ORIGINAL set pattern dance (Skating)" OR DE "PROFESSIONAL skating" OR DE "SPEED skating" OR DE "SKATEBOARDING" OR DE "SNOWMOBILING"OR DE "SLEDDING" OR DE "SKIS & skiing" OR DE "SNOWBOARDING" OR DE "SOCCER" OR DE "TRACK & field" OR DE "ALL-around (Track & field)" OR DE"BOUNDING" OR DE "COLLEGE track & field" OR DE "DECATHLON" OR DE "HEPTATHLON" OR DE "HURDLING (Track & field)" OR DE "INDOOR track & field" ORDE "JAVELIN throwing" OR DE "JUMPING" OR DE "PENTATHLON" OR DE "RUNNINGraces" OR DE "STEEPLECHASING (Track & field)" OR DE "VAULTING" OR DE "WALKING (Sports)" OR DE "WEIGHT throwing" OR DE "WHEELCHAIR track & field" ORDE "WOMEN'S track & field" OR DE "VOLLEYBALL" OR DE "SURFING" OR DE "ROWING" OR DE "POLO" OR DE "KAYAKING" OR DE "CANOES & canoeing" OR DE "BOATS & boating" OR DE "RUGBY football" OR DE "WRESTLING" OR DE "FOOTBALL" OR DE "GOLF" OR DE "CRICKET (Sport)" OR DE "CYCLING" OR DE "BICYCLE racing"OR DE "BICYCLE touring" OR DE "MOTORCYCLING" OR DE "MOUNTAIN biking" OR DE "NIGHT cycling" OR DE "RAILBIKING" OR DE "STUNT cycling" OR DE "URBAN cycling" OR DE "DANCE" OR DE "AERIAL dance" OR DE "AEROBIC dancing" OR DE "BALLET" OR DE "BALLROOM dancing" OR DE "BELLY dance" OR DE "BREAK dancing"OR DE "CHA-cha (Dance)" OR DE "COUNTRY dancing" OR DE "DANCE for

	people with disabilities" OR DE "FLAMENCO" OR DE "FOLK dancing" OR DE "FREE skating" OR DE "HIP-hop dance" OR DE "ICE dancing" OR DE "JAZZ dance" OR DE "LINE dancing" OR DE "LION dance" OR DE "MODERN dance" OR DE "ORIGINAL set pattern dance (Skating)" OR DE "POLE dancing" OR DE "ROUND dancing" OR DE "SALSA (Dance)" OR DE "SHISHIMAI (Dance)" OR DE "STEP dancing" OR DE "TANGO (Dance)" OR DE "TAP dancing" OR DE "WEIGHT lifting" OR DE "BENCH press" OR DE "DEAD lift (Weight lifting)" OR DE "POWERLIFTING" OR DE "SQUAT (Weight lifting)" OR DE "WEIGHT liftingcompetitions" OR DE "JOGGING" OR DE "SNOW & ice climbing" OR DE "ROCK climbing"OR DE "CRACK climbing" OR DE "FACE climbing" OR DE "FREE climbing" OR DE "INDOOR rock climbing" OR DE "INDOOR rock climbing" OR DE "STAIR climbing" OR DE"ROCK climbing training" OR DE "MOUNTAINEERING" OR DE "AEROBIC exercises" OR DE "AEROBIC dancing" OR DE "AEROBICS point system" OR DE "CONTINUOUS training (Exercise)" OR DE "INTERVAL training" OR DE "JUMPING rope" OR DE "KICKBOXING aerobics" OR DE "LOW impact aerobic exercises" OR DE "REBOUNDING (Exercise)" OR DE "RUNNING" OR DE "SPINNING (Exercise cycling)" OR DE "STEP aerobics" OR DE "SWIMMING" OR DE "TAE-Bo (Trademark)" OR DE "WALKING" OR DE "WATER aerobics" OR DE "PHYSICAL training & conditioning" OR DE "ALTITUDE training" OR DE "ANAEROBIC training" DE "PRACTICE (Sports)" OR DE "STRETCH (Physiology)" OR DE "STATIC stretching (Physiology)" OR DE "STRETCH reflex" OR DE"CALISTHENICS" OR DE "HOOP exercises" OR DE "WAND exercises" OR DE "GYMNASTICS" OR DE "ACROBATICS" OR DE "ARTISTIC gymnastics" OR DE "CARTWHEELS" OR DE "HANDSPRINGS" OR DE "HANDSTANDS" OR DE "HEADSTANDS" OR DE "PYRAMIDS (Gymnastics)" OR DE "SOMERSAULTS" OR DE "SWEDISH gymnastics" OR DE "SWIMNASTICS" OR DE "TEAM aerobics" OR DE "TRAMPOLINES" OR DE "TUMBLING" OR DE "PHYSICAL fitness" OR DE "BODYBUILDING" OR DE "CARDIOPULMONARY fitness" OR DE "CARDIOVASCULAR fitness" OR DE "CIRCUIT training" OR DE "COMPOUND exercises" OR DE "EXERCISEtolerance" OR DE "ISOLATION exercises" OR DE "LIANGONG" OR DE "MUSCLE strength" OR DE "PERIODIZATION training" DE "SPORT for all" OR DE "PHYSICAL activity" OR DE "EXERCISE" OR DE "ABDOMINAL exercises" OR DE "AEROBIC exercises" OR DE "ANAEROBIC exercises" OR DE "AQUATIC exercises" OR DE "ARM exercises" OR DE "BACK exercises" OR DE "BREATHING exercises" OR DE "BREEMA" OR DE "BUTTOCKS exercises" OR DE "CHAIR exercises" OR DE "CHEST exercises" OR DE "CIRCUIT training" OR DE "COMPOUND exercises" OR DE "COOLDOWN" OR DE "DO-in" OR DE "FACIAL exercises" OR DE "FALUN gong exercises" OR DE "FOOT exercises" OR DE "HAND exercises" OR DE "HATHA yoga" OR DE "HIP exercises" OR DE "ISOKINETIC exercise" OR DE "ISOLATION exercises" OR DE "ISOMETRIC exercise"OR DE "ISOTONIC exercise" OR DE "KNEE exercises" OR DE "LEG exercises" OR DE "LIANGONG" OR DE "MULAN quan" OR DE "MUSCLE strength" OR DE "PILATES method" OR DE "PLYOMETRICS" OR DE "QI gong" OR DE "REDUCING exercises" ORDE "RUNNING" OR DE "SEXUAL exercises" OR DE "SHOULDER exercises" OR DE"STRENGTH training" OR DE "STRESS management exercises" OR DE "TAI chi" OR DE "TREADMILL exercise" OR DE "WHEELCHAIR workouts" OR DE "YOGA" OR DE

	"WARMUP" OR DE "AQUATIC sports" OR DE "BALL games" OR DE "BASEBALL" OR DE "HOCKEY" OR DE "LOG-chopping (Sports)" OR DE "RACKET games" OR DE "ROLLER skating" OR DE "SCHOOL sports" OR DE "SOFTBALL" OR DE "SPORTS competitions" DE "WINTER sports" OR DE "AERONAUTICAL sports" OR DE "COLLEGE sports" OR DE "CONTACT sports" OR DE "CROSS-training (Sports)" OR DE "DISC golf" OR DE "ENDURANCE sports" OR DE "EXTREME sports" OR DE "FANTASY sports" OR DE"GAELIC games" OR DE "GOODWILL Games" OR DE "INDIVIDUAL sports" OR DE "KNIFE throwing" OR DE "MILITARY sports" OR DE "OLYMPIC Games" OR DE "PARKOUR" OR DE "PROFESSIONAL sports" OR DE "RACING" OR DE "RACKET games" OR DE "RODEOS" OR DE "SENIOR Olympics" OR DE "SHOOTING (Sports)" OR DE "TEAM sports" OR TI (Exercise* OR physical-activit* OR Gymnastic* OR Calisthenic* OR stretch* OR train* OR condition* OR run* OR jog* OR swim* OR walk* OR climb* OR weight-lift* OR Ambulat* OR Physical-Exertion OR physical-effort OR Fitness OR pilate* OR qigong OR qi-gong OR ch'i-kung OR danc* OR tai-ji OR tai-chi OR tai-ji-quan OR taiji OR taijiquan OR t'ai-chi OR tai-chi-chuan OR yoga OR Sport* OR athletic* OR baseball* OR softball* OR basketball* OR netball* OR bicycling OR cycling OR boxing* OR cricket OR football* OR rugb* OR golf* OR hockey* OR wrestl* OR martial-art* OR hap-di-do OR judo OR karate OR jujitsu OR tae-kwon-do OR aikido OR wushu OR kung-fu OR gong-fu OR gongfu OR mountaineer* OR tennis OR Racquetball OR Racketball OR Racket-Ball OR Badminton OR Lacrosse OR skating* OR skateboard* OR snowmobiling OR sledding OR skiing OR snowboard* OR soccer* OR track* OR volleyball* OR surfing OR rowing ORpolo OR kayaking OR canoeing OR boating OR surfboarding OR recreation* OR garden* OR ballet OR hip-hop OR jazz OR tap OR salsa) OR AB (Exercise* OR physical-activit* OR Gymnastic* OR Calisthenic* OR stretch* OR train* OR condition* OR run* OR jog* ORswim* OR walk* OR climb* OR weight-lift* OR Ambulat* OR Physical-Exertion OR physical-effort OR Fitness OR pilate* OR qigong OR qi-gong OR ch'i-kung OR danc* OR tai-ji OR tai-chi OR tai-ji-quan OR taiji OR taijiquan OR t'ai-chi OR tai-chi-chuan OR yoga OR Sport* OR athletic* OR baseball* OR softball* OR basketball* OR netball* OR bicyclingOR cycling OR boxing* OR cricket OR football* OR rugb* OR golf* OR hockey* OR wrestl* OR martial-art* OR hap-di-do OR judo OR karate OR jujitsu OR tae-kwon-do OR aikido ORwushu OR kung-fu OR gong-fu OR gongfu OR mountaineer* OR tennis OR Racquetball OR Racketball OR Racket-Ball OR Badminton OR Lacrosse OR skating* OR skateboard* OR snowmobiling OR sledding OR skiing OR snowboard* OR soccer* OR track* OR volleyball* OR surfing OR rowing OR polo OR kayaking OR canoeing OR boating OR surfboarding OR recreation* OR garden* OR ballet OR hip-hop OR jazz OR tap OR salsa))<br><br>refs 57
CENTRAL	Search Name: Lowman CF Exercise Body Composition<br>Date Run: 04/01/2021 18:07:12<br>Comment:<br>ID Search Hits

	#1 MeSH descriptor: [Cystic Fibrosis] explode all trees 1805<br>#2 (Cystic-Fibrosis OR Mucoviscidosis OR Fibrocystic-Disease*):ti,ab,kw 6134<br>#3 #1 OR #2 6134<br>#4 MeSH descriptor: [Exercise] explode all trees 24678<br>#5 MeSH descriptor: [Exercise Therapy] explode all trees 13850<br>#6 MeSH descriptor: [Physical Exertion] explode all trees 3851<br>#7 MeSH descriptor: [Recreation] explode all trees 16941<br>#8 (Exercise* OR Physical-Exertion* OR physical-effort* OR physical-activit* OR Gymnastic* OR Calisthenic* OR stretch* OR train* OR run OR runs OR running OR jog OR jogs OR jogging OR swim* OR walk* OR climb* OR weight-lift* OR Ambulat* OR Physical-Fitness OR pilate* OR qigong OR qi-gong OR ch'i-kung OR danc* OR tai-ji OR tai-chi OR tai-ji-quan OR taiji OR taijiquan OR t'ai-chi OR tai-chi-chuan OR yoga OR Sport* OR athletic* OR baseball* OR softball* OR basketball* OR netball* OR bicycling OR cycling OR boxing* OR cricket OR football* OR rugb* OR golf* OR hockey* OR wrestl* OR martial-art* OR hap-di-do OR judo OR karate OR jujitsu OR taekwon-do OR aikido OR wushu OR kung-fu OR gong-fu OR gongfu OR mountaineer* OR tennis OR Racquetball OR Racketball OR Racket-Ball OR Badminton OR Lacrosse OR skating* OR skateboard* OR snowmobiling OR sledding OR skiing OR snowboard* OR soccer* OR track OR volleyball OR surfing OR rowing OR polo OR kayaking OR canoeing OR boating OR surfboarding OR recreation* OR garden* OR ballet OR hip-hop OR jazz OR tap OR salsa OR Sport*):ti,ab,kw<br>252580<br>#9 #4 OR #5 OR #6 OR #7 OR #8 253260<br>#10 MeSH descriptor: [Physical Appearance, Body] explode all trees 294<br>#11 MeSH descriptor: [Body Composition] explode all trees 5124<br>#12 MeSH descriptor: [Body Weights and Measures] explode all trees 32195<br>#13 MeSH descriptor: [Organ Size] explode all trees 943<br>#14 #12 NOT #13 31252<br>#15 (physical-appearance OR outward-appearance OR "Body Composition" OR body-measure* OR Body-weight OR body-mass-index OR waist-circumference OR weight-loss OR weight-gain):ti,ab,kw 102161<br>#16 #10 OR #11 OR #14 OR #15 109429<br>#17 #3 AND #9 AND #16 119
